# Geochemical Effects of Groundwater Interaction with Steel Slag Aggregate Used in Road Construction

**DOI:** 10.3390/ma19163457

**Published:** 2026-08-14

**Authors:** Zdzisław Adamczyk, Aleksandra Czajkowska, Barbara Białecka, Magdalena Cempa

**Affiliations:** 1 Faculty of Mining, Safety Engineering and Industrial Automation, Silesian University of Technology, Ul. Akademicka 2, 44-100 Gliwice, Poland; aleksandra.czajkowska@polsl.pl; 2 Department of Environmental Analysis and Circular Economy Technologies, Central Mining Institute—National Research Institute, Plac Gwarków 1, 40-166 Katowice, Poland; bbialecka@gig.eu (B.B.); mcempa@gig.eu (M.C.)

**Keywords:** steel slag aggregate, groundwater interaction, inverse geochemical modelling, PHREEQC, secondary mineralisation

## Abstract

This study assessed the effects of groundwater interacting with aggregate produced from steel slag, used as a ballast material in a waterproofing system protecting the road surface under high groundwater table conditions. Field investigations were carried out, with hydrochemical analyses of groundwater and water draining through the structure, mineralogical characterisation of the slag, and geochemical modelling using the PHREEQC programme with inverse modelling. The results showed that water flow through the slag aggregate caused strong alkalisation of the solution and changes in the concentrations of Ca, Mg, Na, Cl, sulphates and carbonate components. Inverse modelling enabled the identification of eight acceptable mass balance models. The main primary phases involved in the transformations were larnite, merwinite, mayenite, halite and, locally, slag glass. Their dissolution contributed Ca, Mg, Al, Si, Na and Cl to the solution. The increases in Na and Cl concentrations were interpreted primarily as the result of an external influx of road salt, rather than as an intrinsic property of the slag. The secondary products were dominated by amorphous silica, calcite, ettringite and brucite, indicating silica removal, carbonation, sulphate fixation and the partial immobilisation of Mg. The results confirm that slag aggregate remains geochemically active in contact with groundwater; however, simultaneous carbonation and secondary mineralisation favour the gradual stabilisation of the water–slag system.

## 1. Introduction

In recent decades, there has been growing interest in the use of industrial by-products in infrastructure construction. This interest is particularly significant in the context of the circular economy and the reduction in industrial waste disposal. One of the most commonly used materials of this type is metallurgical slag produced during the manufacture of iron and steel. Following appropriate technological preparation, these materials can be used as aggregate in road construction and in engineering embankments, as well as for ballast or drainage in hydrotechnical and road structures. The use of metallurgical slag as a substitute for natural aggregates helps to reduce the exploitation of mineral resources and minimise the amount of waste sent to landfill, which is of significant environmental and economic importance [1,2,3,4,5,6,7].

Metallurgical slags are characterised by a complex mineral and chemical composition, resulting from the technological conditions of the metallurgical process and the type of raw materials used. Their composition is dominated by calcium and magnesium silicates and aluminosilicates, as well as iron phases and an glassy amorphous phase. The most common phases include, amongst others, merwinite, monticellite, akermanite and gehlenite, as well as various spinel phases [8]. At the same time, slags may contain potentially mobile elements such as chromium, vanadium, molybdenum or zinc, which, under certain geochemical conditions, may leach into the aquatic environment [2,4,9,10,11]. For this reason, assessing the impact of metallurgical slags on the aquatic environment is an important aspect of research into their use in civil engineering structures.

A particularly important issue is the behaviour of slags under conditions of prolonged contact with groundwater. Under such conditions, complex geochemical processes take place, including the dissolution of primary mineral phases, the hydration of reactive components, and the secondary crystallisation of minerals formed as a result of the interaction between the material and water [12,13]. These processes can affect both the durability of the material and the chemical composition of the infiltrating water [14]. These processes often lead to the formation of strongly alkaline leachates, the chemical composition of which is governed by the equilibrium between the solid phases and the aqueous solution [9,15,16]. The high pH values observed in waters in contact with slag are usually associated with the dissolution of calcium-rich phases, such as free CaO, portlandite or reactive calcium silicates. In a subsequent stage of the system’s geochemical evolution, secondary minerals are precipitated, in particular calcite, hydrotalcite and other carbonate and hydroxide phases, which may limit the mobility of certain trace elements [15,16,17,18,19].

Studies on the leaching of constituents from metallurgical slags indicate that this process is strongly dependent on factors such as pH, redox potential, the chemical composition of the water, and the water–rock contact time [9,17]. In many cases, an initial increase in the alkalinity of water in contact with slags is observed, resulting from the dissolution of calcium phases, followed by a gradual stabilisation of the chemical composition due to the precipitation of secondary minerals [14]. Understanding these processes requires the integration of mineralogical and chemical data with geochemical modelling.

In recent years, geochemical modelling tools have been used increasingly frequently in research into the interaction of waste materials with water. One of the most widely used programmes in this field is PHREEQC, developed by the US Geological Survey [20,21]. This programme enables the simulation of mineral dissolution and precipitation processes, ionic reactions in aqueous solutions, and the transport of chemical constituents [13,14,22]. A particularly useful tool is the inverse modelling module, which allows the identification of geochemical reactions responsible for observed changes in the chemical composition of water between successive sampling points [20,23]. This method is widely used in hydrogeochemical studies, including the analysis of the impact of anthropogenic materials on the aquatic environment.

Despite numerous laboratory studies on the leaching of constituents from metallurgical slags, relatively few investigations have examined slag aggregates incorporated into actual engineering structures and exposed to groundwater an extended period. Field studies are particularly important because they account for the combined effects of groundwater flow, seasonal hydrochemical variability, external solute inputs and interactions with the surrounding construction materials. Consequently, laboratory tests do not always fully reproduce the geochemical processes occurring under field conditions [17]. Moreover, the hydrochemical effects of slag–water interaction and the mineralogical transformations of the slag are often considered separately, making it difficult to identify the particular reactions responsible for the observed changes in water chemistry.

From the perspective of engineering practice, it is therefore important to determine how prolonged contact between metallurgical slag and groundwater affects both the phase composition of the aggregate and the quality of water infiltrating through the structure. The integration of field hydrochemical observations with mineralogical data and inverse geochemical modelling may provide a more comprehensive assessment than conventional leaching tests alone. In particular, it allows the observed changes in water chemistry to be related to the dissolution of primary slag phases, the precipitation of secondary minerals and possible external inputs of dissolved constituents. This knowledge is relevant to the environmental assessment and design of road structures containing slag aggregate under conditions of high groundwater table.

The aim of this study was to identify and quantitatively characterise the geochemical processes occurring during the long-term interaction of groundwater with aggregate produced from steelmaking slag used as ballast material in a waterproofing system protecting a road pavement. Particular attention was paid to the effect of water–slag contact on the chemical composition of water draining through the structural layers and to the mineralogical transformations of the slag, including the dissolution of reactive primary phases and the formation of secondary mineral products. The novelty of the study lies in combining observations from a full-scale road structure with mineralogical characterisation and PHREEQC inverse modelling to identify and quantify the mineral mass transfers consistent with the measured changes in water chemistry. This integrated approach also enables the intrinsic effects of slag–water interaction to be distinguished from external influences, particularly the influx of Na and Cl associated with the use of road de-icing salts.

Groundwater and water draining through the structure were subjected to chemical analysis, while the slag aggregate was characterised mineralogically. These data were subsequently integrated using PHREEQC Inverse Modeling to determine the direction and magnitude of the mineral mass transfer responsible for the observed evolution of the water–slag system.

## 2. Research Area

**Figure 1:**
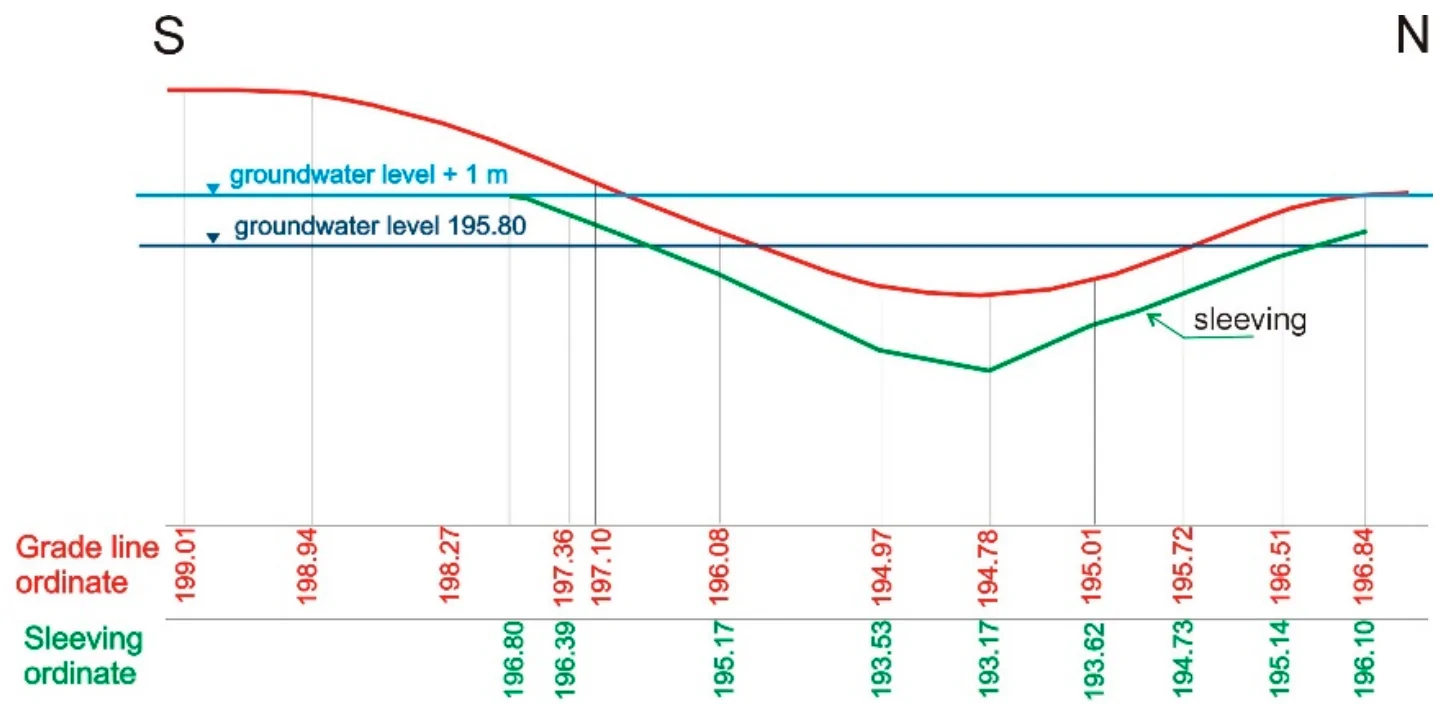
A sketch showing the lowering of the road profile grade line below the groundwater table in the study area.

The research area was located in southern Poland, within an urbanised area undergoing transport infrastructure redevelopment. As part of a project to modernise the railway line, the road network in the vicinity of the railway viaduct was rebuilt. This work involved lowering the road surface below the level of the groundwater table (Figure 1).

In order to protect civil engineering structures, including viaducts and road infrastructure elements, waterproofing was carried out comprising a protective insulation layer and a surface drainage system for rainwater infiltrating into the structure. Steel slag was used as ballast to fill the structure (the so-called trough) due to its high unit weight. The producer and the specific steelmaking process from which the slag originated could not be identified from the available information, as these details were not disclosed by the construction contractor due to commercial confidentiality. Therefore, the material was characterised on the basis of its experimentally determined chemical and mineralogical composition.

After several years of operation, damage to the road surface was observed, as well as the presence of white deposits on the surfaces of wells and stormwater inlets within the waterproofing system.

## 3. Research Methodology

### 3.1. Groundwater and Drainage Water Testing

**Figure 2:**
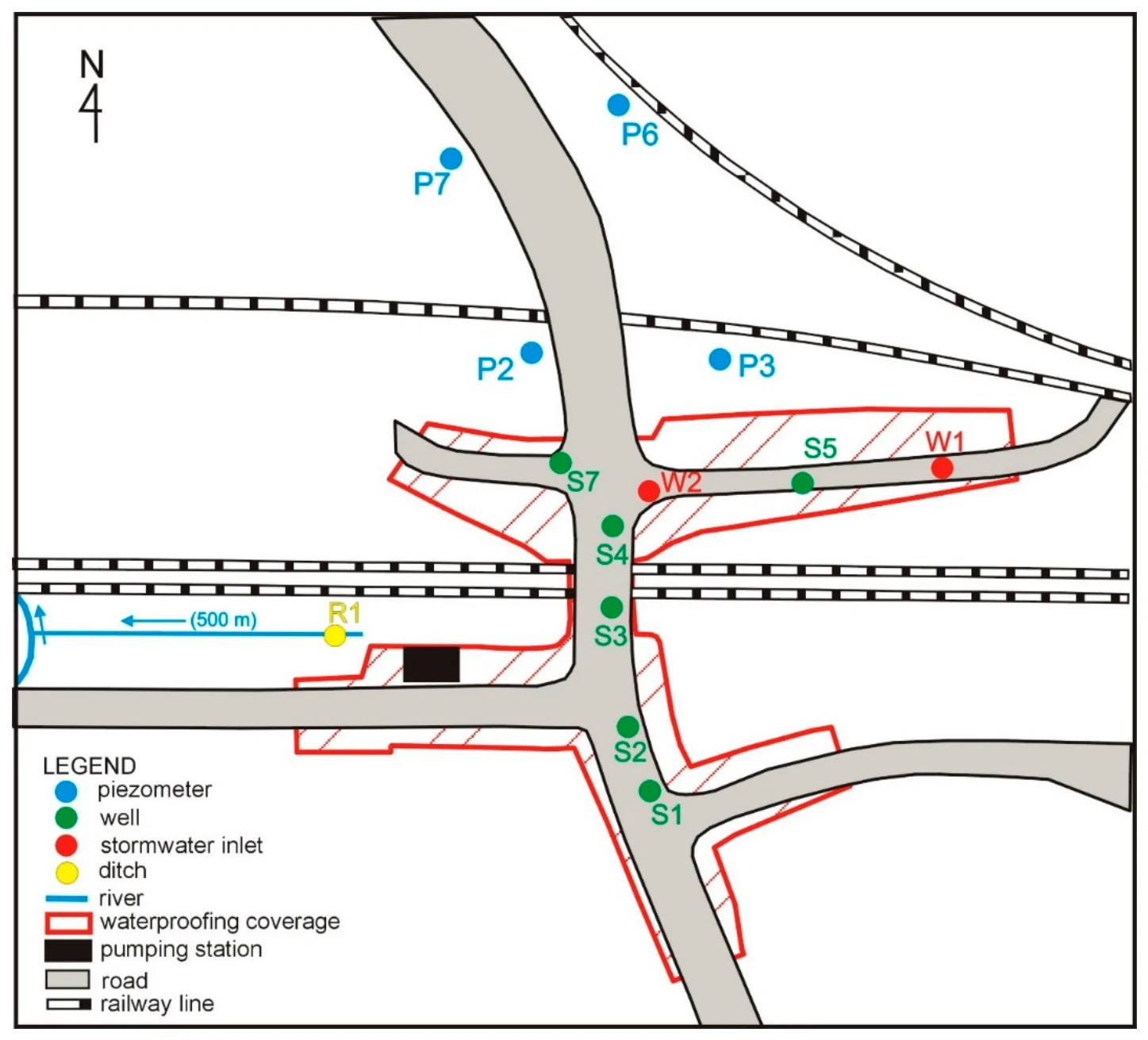
Location of water sampling points.

During the winter, groundwater samples were taken at 12 locations within the study area, comprising 4 piezometers (P2, P3, P6 and P7), 6 wells (S1, S2, S3, S4, S5 and S7) and 2 stormwater inlets (W1 and W2) (Figure 2). A surface water sample was also collected from a ditch (R1), which carries drainage water from the waterproofing system, as well as rainwater and surface runoff, to the neighbouring river.

The scope of the tests included the determination of colour, pH, mineralisation, total hardness, carbonate hardness, non-carbonate hardness, calcium hardness and magnesium hardness, alkalinity, chlorides, sulphates, bicarbonates, calcium, magnesium, sodium, potassium, iron, manganese, aluminium and silicon. The analyses were carried out using conventional laboratory methods (titration and gravimetric methods), as well as the Elmetron CX-401 multifunctional measuring instrument (Elmetron, Zabrze, Poland), the Slandi LF 300 microprocessor-based photometer (Slandi Sp. z o.o., Michałowice, Poland) and the ICP-AES JY 2000 spectrometer (Jobin Yvon, Longjumeau, France).

### 3.2. Phase Composition of Slag

Slag aggregate samples were collected during the winter season, concurrently with water sampling, at locations outside the roadway and in the immediate vicinity of water-sampling points P3 and S5. The samples were taken from a depth of approximately 2.0–2.5 m, while maintaining a sufficient distance from the bottom waterproofing layer to avoid damaging it. One aggregate sample weighing approximately 2 kg was collected at each location.

The phases present in the slag samples were identified using X-ray diffraction (XRD). The measurements were carried out on a Bruker Phaser 2 diffractometer, equipped with a copper (CuK_α_) X-ray tube, at a voltage of 30 kV and a current of 10 mA. The diffractograms were recorded in the 2theta angular range from 8° to 72°. The DIFFRACT.EVA software (v. 6.1.0.4) and the PDF-5+ 2025 database were used for phase identification. Quantitative phase analysis was performed using the Rietveld method, whilst the proportion of X-ray-amorphous or weakly crystalline material was determined using an internal ZnO standard.

The phase characterisation of the slags was supplemented by micro-area analyses using scanning electron microscopy (SEM) coupled with X-ray microanalysis (EDS). The investigations were carried out using an SU3500 scanning electron microscope (Hitachi High-Technologies Corporation, Tokyo, Japan), equipped with a Thermo Scientific UltraDry X-ray energy-dispersive spectrometer operating with the NORAN System 7 (Thermo Fisher Scientific, Madison, WI, USA). The EDS analyses enabled the chemical composition of selected micro-areas to be determined, including the relict glassy phase of the slag, and also aided in the identification of the crystalline phases observed in the SEM images.

### 3.3. Geochemical Modelling

An inverse model was developed for the geochemical processes occurring during the contact between groundwater and steel slag, using the INVERSE_MODELING module of the PHREEQC v. 3.0 programme. This enables the quantitative reconstruction of reaction pathways based on the chemical composition of the water before and after contact with the material. This module identifies sets of reactions involving dissolution, precipitation, ion exchange and solution mixing, which lead to the observed changes in the chemistry of the water. This is particularly useful in studies of interactions within the water–slag system.

**Figure 3:**
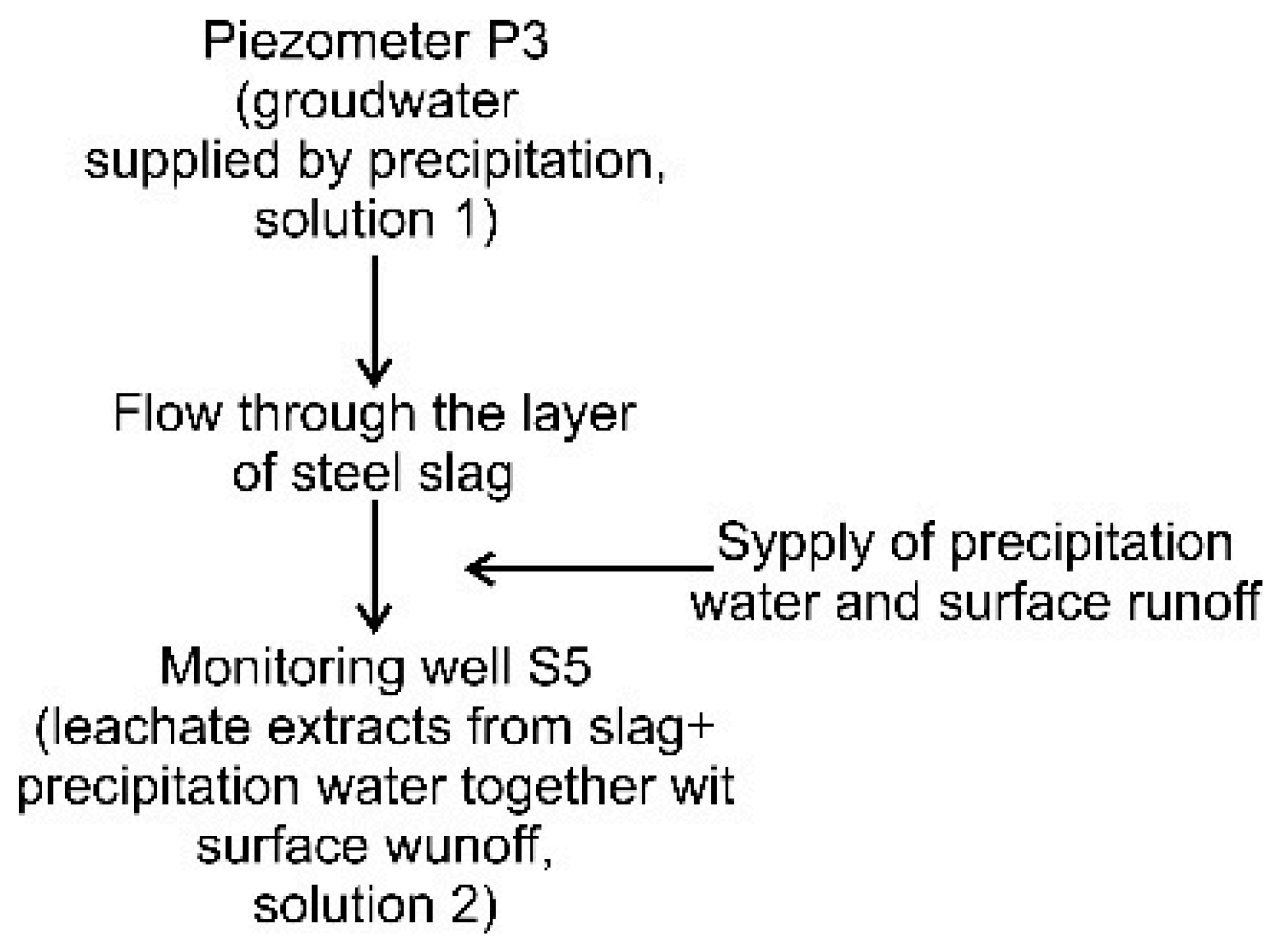
Schematic representation of water flow through waterproofing layer containing a steel slag layer.

Real-world data obtained from laboratory water tests were used to construct the model. The diagram (Figure 3) illustrates the flow pattern of water through the waterproofing system with a layer of steelmaking slag, which acts as dead load on the structure. The input data for the model were as follows: (i) the initial solution (solution 1)—groundwater sampled from piezometer P3, representing the natural chemical composition of the Quaternary aquifer; (ii) the final solution (solution 2)—water from well No. 5 following contact with the slag material (Figure 2); (iii) the phase composition of the mineral system taken into account in the calculations.

Inverse geochemical modelling was performed using the INVERSE_MODELING functionality of PHREEQC version 3. The mathematical formulation of inverse modelling in PHREEQC is based on simultaneous mole-balance equations for the aqueous components, solid phases and, where applicable, redox reactions and water [20]. Its implementation and input structure in PHREEQC version 3 are described by Parkhurst and Appelo [21].

In the present study, the model comprised one initial solution (P3), one final solution (S5), and the transfer of the selected solid phases. Therefore, the general mole-balance formulation of Parkhurst and Appelo [20] can be represented for the investigated two-solution system by the following balance for each element *i*:

(1) $$\left(T_{i,S5}+\delta_{i,S5}\right)-\alpha_{P3}\left(T_{i,P3}+\delta_{i,P3}\right)=\;\overset{n}{\underset{j=1}{\sum }}v_{ij}\Delta n_{j}$$

where *T_i,P_*_3_ and *T_i,S_*_5_ are the total amounts of element *i* in the initial and final solutions, respectively; *δ_i,P_*_3_ and *δ_i,S_*_5_ are adjustments to the respective analytical values constrained by the uncertainty limits specified in the inverse model; *α_P_*_3_ is the model-calculated fraction of the initial solution contributing to one unit of the final solution; *ν_ij_* is the stoichiometric coefficient of element *i* in the dissolution reaction of phase *j*; and Δ*n_j_* is the net mole transfer of phase *j*. Because phase reactions in PHREEQC are conventionally written as dissolution reactions, positive values of Δ*n_j_* indicate dissolution, whereas negative values indicate precipitation [20,21]. The amounts of aqueous components and phase transfers were normalised to a common calculation basis of 1 kg of water, and the phase transfers are therefore reported in mol kg^−1^ H_2_O.

The applied inverse model represents an endpoint mole balance between solutions P3 and S5 and does not constitute a kinetic or reactive-transport simulation [20,21]. Consequently, the physical water-to-slag ratio and contact time were not input parameters of the calculation. These field-scale parameters could not be independently constrained because the road structure constitutes an open and spatially heterogeneous groundwater-flow system. Neither the mass of slag effectively contacted by the sampled water nor the groundwater flow path, velocity and residence time were monitored. Accordingly, the calculated phase transfers represent the amounts of phases required per kilogram of water to satisfy the elemental balances within the specified uncertainty limits, rather than reaction rates or transfers associated with a known mass of slag and a defined contact period.

The inverse modelling was based on the following assumptions and constraints controlling the mass balance and permissible geochemical pathways:

- The selection of mineral phases permitted for dissolution and precipitation, with particular emphasis on phases characteristic of steelmaking slag;
- The determination of uncertainty ranges for the elemental balance to ensure solutions consistent with the results of chemical analyses of the water;
- Taking into account and modifying ion-exchange reaction schemes to assess their impact on closing the mass balance;
- Imposing constraints on the masses of phases undergoing dissolution and precipitation, ensuring that the calculations are consistent with the observed mineralogy of the pre- and post-reaction systems;
- Controlling the contribution of secondary phases, in particular those responsible for the immobilisation of trace metals;
- Iteratively fitting inverse models to the ion balance by testing alternative sets of reactions until agreement is achieved between the calculation results and the chemical composition of the water, as well as the observed mineral transformations.

The calculations were carried out using the phreeqc_rates.dat thermodynamic database, selected because it provides thermodynamic definitions for the mineral phases identified experimentally in the investigated slag. The inverse calculations were therefore constrained by the mineral assemblage determined by XRD and SEM-EDS analyses, rather than by hypothetical phase combinations. The acceptance of the model solutions was based on the criterion of mass balance closure and consistency with the observed sequence of mineral transformations.

These assumptions and constraints influence the number and composition of the accepted models and the calculated phase transfers, thereby contributing to the non-uniqueness of the inverse solutions.

## 4. Results

### 4.1. Chemical Composition of the Water

Groundwater samples taken from piezometers located outside the waterproofing system reflected the natural chemical composition of the Quaternary aquifer in this region. The water was pale straw-coloured, with a slightly acidic pH (pH 6.81–6.96), classified as hard water (total hardness 20.19–26.36 °n) and with a mineralisation ranging from 664.46 mg/dm^3^ (piezometer P7) to 902.5 mg/dm^3^ (piezometer P3). The chemical composition was dominated by bicarbonates (average 357.48 mg HCO_3_/dm^3^) and calcium (average 101.2 mg Ca/dm^3^). In piezometers P2 and P3, situated closer to the railway viaduct (Figure 2), a higher sulphate content (average 187.5 mg SO_4_/dm^3^) was observed compared with piezometers P6 and P7, which were further away from the viaduct (average 89 mg SO_4_/dm^3^). The highest concentration of iron, amounting to 0.294 mg Fe/dm^3^, was observed in the water from piezometer P7, whilst the highest concentration of manganese—0.38 mg Mn/dm^3^—was found in the water from piezometer P6 (Table 1).

The water from the wells consisted of a mixture of drainage water from the waterproofing system, rainwater and surface runoff. The water varied significantly in terms of chemical composition, depending on the location of the well. The water from wells S1, S2 and S3 (Figure 2), which had a rusty-brown colour, was slightly acidic (pH 6.59–6.96) and had a mineralisation range of 889.22 mg/dm^3^ (well S2) to 1242.56 mg/dm^3^ (well S1). These were very hard waters (26.92–33.09 °n), in which bicarbonates (mean 462.44 mg HCO_3_/dm^3^), calcium (average 140.28 mg Ca/dm^3^) and sulphates (average 227.67 mg SO_4_/dm^3^) were the predominant compounds. The iron content ranged from 0.209 mg Fe/dm^3^ (well S1) to 1.411 mg Fe/dm^3^ (well S3), and the manganese content ranged from 0.05 mg Mn/dm^3^ (well S1) to 0.33 mg Mn/dm^3^ (well S2) (Table 1).

The water collected from wells S4, S5 and S7 (Figure 2) was alkaline (pH 12.49–12.96), had high mineralisation (1770.26–3919.55 mg/dm^3^) and was dark in colour—ranging from grey to black. These waters had very high total hardness, ranging from 83 °n in well S4 to 155.9 °n in well S5, with a predominance of carbonate and calcium hardness. The average calcium ion content in the water samples taken from wells S4, S5 and S7 was 619.9 mg Ca/dm^3^ and was more than four times higher than the average calcium ion content in wells S1, S2 and S3. At the same time, in the wells draining the northern part of the waterproofing system, a low sulphate content was found (average 63.67 mg SO_4_/dm^3^); more than three times lower than in the water from the southern section. The iron content in the water from wells S4, S5 and S7 ranged from 0.05 to 0.364 mg Fe/dm^3^, and the manganese content was 0.05 mg Mn/dm^3^ (Table 1).

The water samples taken from stormwater inlets W1 and W2 consisted of a mixture of rainwater and surface runoff (Figure 2). The water was slightly yellowish (W2) and colourless (W1), and the pH was slightly alkaline (pH 7.17–8.18). The concentration of dissolved constituents ranged from 700.23 mg/dm^3^ (W2) to 837.5 mg/dm^3^ (W1).

The water from stormwater inlet W2 was dominated by sodium ions (125.28 mg Na/dm^3^) and sulphates (199.0 mg SO_4_/dm^3^), whilst the water from stormwater inlet W1 was dominated by calcium (120.24 mg Ca/dm^3^) and bicarbonates (359 mg HCO_3_/dm^3^). The average iron content was 0.11 mg Fe/dm^3^, and that of manganese was 0.065 mg Mn/dm^3^ (Table 1).

The water from the surface ditch, which carries drainage water from the waterproofing system (R1), rainwater and water from street drainage into the river (Figure 2), had an alkaline pH (pH 12.45) and a mineralisation of 1249.34 mg/dm^3^. The water was grey-brown in colour. Its chemical composition was dominated by bicarbonate ions (766.67 mg HCO_3_/dm^3^), chlorides (369.2 mg Cl/dm^3^) and calcium (232.46 mg Ca/dm^3^) (Table 1).

### 4.2. Phase Composition of Slag

**Figure 4:**
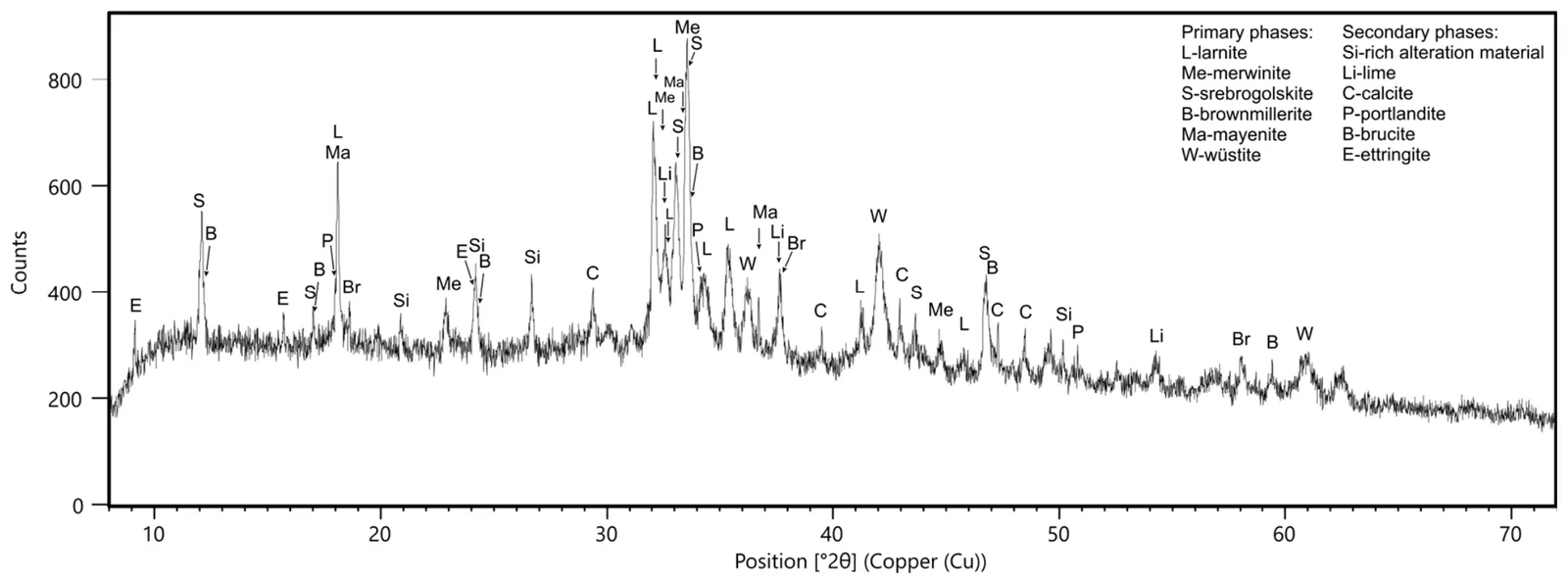
X-ray diffraction pattern of the slag sample.

In the slag under investigation, alongside primary phases such as wüstite, srebrodolskite, brownmillerite, mayenite, larnite and merwinite, the presence of secondary phases was identified, including lime, calcite, portlandite, brucite and ettringite (Figure 4). These secondary phases are thought to result from hydration and carbonation reactions of the slag’s primary constituents occurring upon contact with water and CO_2_. Furthermore, quantitative analysis using the Rietveld method revealed a significant proportion of X-ray-amorphous or weakly ordered material. However, this fraction should not be interpreted solely as primary slag glass, as it may include both a relict glassy phase and secondary, amorphous or low-crystalline products of the transformation of calcium silicates, particularly silica gels and C-S-H-type products with varying degrees of decalcification. The quantitatively determined content of the amorphous fraction should therefore be treated as the total X-ray-amorphous or weakly crystalline fraction, and not solely as the proportion of primary slag glass. The presence of weak reflections attributed to crystalline silica, similar in position to quartz reflections, should be interpreted as evidence of partial reorganisation or local secondary crystallisation of the silica phase, rather than as proof of the original presence of quartz in the slag.

In terms of quantity, the primary crystalline phases predominated, accounting for 63.5%. The relict glassy phase of the slag accounted for 26.0%, whilst the proportion of secondary phases and secondary amorphous or weakly crystalline products was 10.5% (Table 2).

**Figure 5:**
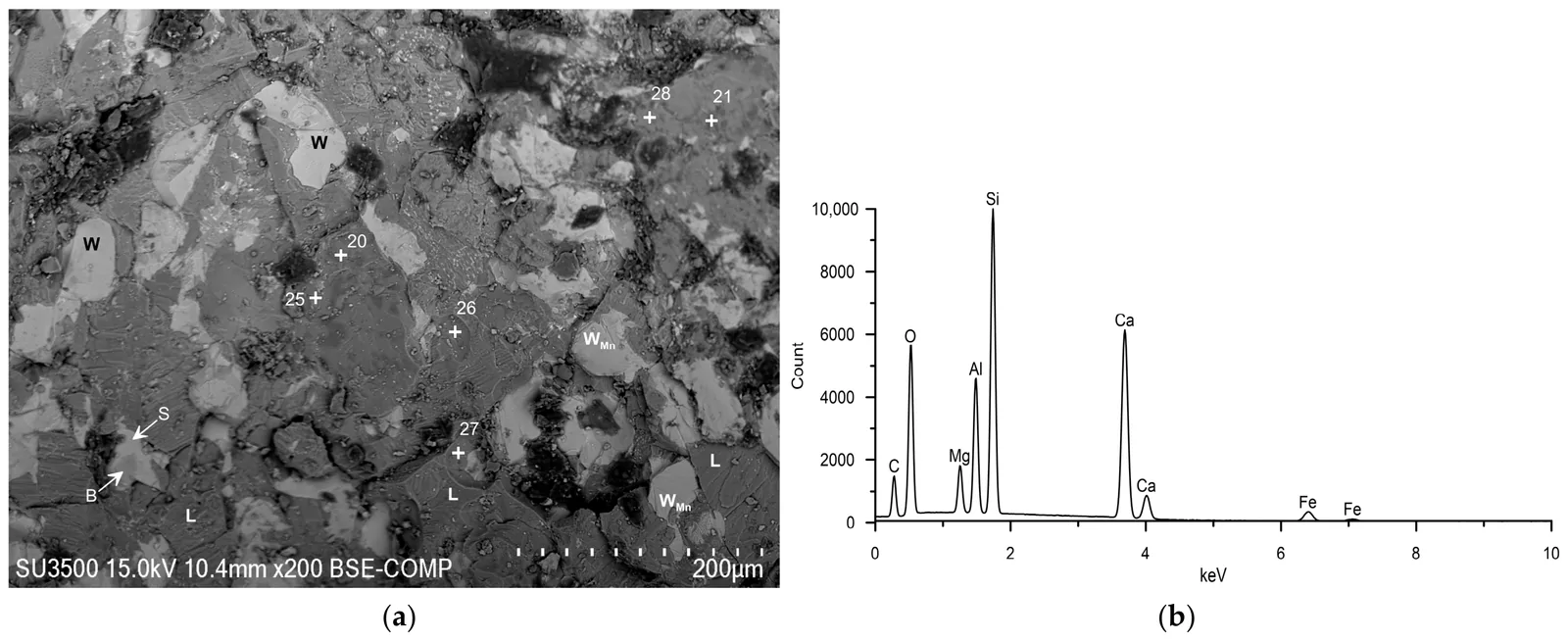
SEM-BSE image and representative EDS spectrum of slag glass in the initial slag sample: (**a**) back-scattered electron SEM image showing the main crystalline phases and selected glassy micro-areas analysed by EDS. Abbreviations: B—brownmillerite; L—larnite; S—spinel; W—wüstite; W_Mn_—Mn-bearing wüstite. The “+” symbols and associated numbers indicate EDS analysis points selected within slag-glass micro-areas; the point numbers correspond to those reported in Table 3 and were used for chemical composition and empirical formula calculations. (**b**) example EDS spectrum of slag glass acquired at measurement point 27.

EDS analyses of selected micro-areas of slag enamel (Figure 5a,b) indicate its relatively homogeneous composition in the Ca-Mg-Al-Si-Fe system. The chemical composition is dominated by SiO_2_ and Al_2_O_3_, with average contents of 39.77% by weight and 33.82% by weight, respectively (Table 3).

CaO and MgO are present in smaller quantities, averaging 18.73% by weight and 5.22% by weight, whilst FeO is a minor component, with an average content of 2.26% by weight. The calculated atomic ratios indicate that the contents of Al and Si are close to one atom per formula unit, whilst Ca and Mg are present in amounts of approximately 0.50 and 0.20 atoms, respectively. On this basis, the average empirical formula for the analysed micro-areas of slag glass can be written as Ca_0.50_Mg_0.20_AlSiFe_0.05_O_4.25_.

It should be emphasised that the empirical formula given refers to selected microareas of slag glass analysed by the EDS method and should not be directly equated with the composition of the entire X-ray-amorphous fraction determined by the XRD method.

## 5. Geochemical Modelling of the Slag–Water System

### 5.1. Experimental Constraints and Conceptual Basis for Inverse Modelling

#### 5.1.1. Hydrochemical Constraints and Solution Components

For geochemical modelling, given the pattern of water flow through the waterproofing system containing a layer of steelmaking slag, water samples from points P3 (solution 1) and S5 (solution 2) were selected. The initial solution (P3) consisted of groundwater, representing the natural chemical composition of the Quaternary aquifer, whilst the final solution (S5) comprised water that had come into contact with the slag material and exhibited the greatest changes in chemical composition. The quality parameters of these waters, calculated using the PHREEQC programme’s logic, served as input data for the model (Table 4). The differences in the chemical composition of the waters from P3 and S5 indicate reactions taking place between solution 1 and the original phase constituents of the slag (Table 2). The P3–S5 pair was selected based on its hydrogeochemical setting and the magnitude of the observed compositional changes. Therefore, the inverse models represent plausible reaction pathways for this specific system rather than a universal groundwater–slag interaction mechanism.

The geochemical evolution of the water, from the initial to the final solution, was accompanied by a marked increase in pH from 6.96 to 12.49, as well as a significant rise in alkalinity and in the concentrations of calcium, sodium and magnesium. At the same time, a significant decrease in sulphate concentration was observed (Table 4).

Such changes in water chemistry are characteristic of leachates from steel slag and indicate the intensive dissolution of reactive phases rich in calcium, leading to the rapid alkalisation of the solution [17,24,25]. Similar trends were observed, amongst others, by Li J. et al. [26], Mayes W.M. et al. [9] and Proctor D.M. et al. [2] in their studies of steelmaking slags, where the dissolution of calcium silicates was the main mechanism responsible for the increases in the pH and alkalinity of the leachate.

#### 5.1.2. Selection and Geochemical Roles of Mineral Phases

The mineral phases were assigned the following statuses: ‘dis’—primary phase permitted to dissolve; ‘pre’—secondary phase permitted to precipitate. The potential geochemical role of each phase within the model system was specified (Table 5).

As the water samples were collected during the winter, the elevated Ba and Cl concentration were most likely associated with the inflow of road salt. This interpretation is supported by the very low Na and Cl concentrations in a groundwater sample collected on the same day from a nearby piezometer (P7) located in a slightly elevated position and probably unaffected by runoff from the roadway. The possible use of an anti-caking agent containing potassium compounds may also explain the potassium contribution. Accordingly, halite (NaCl) and kalicinite (KHCO_3_) were included in the set of phases to achieve mass balance closure for sodium, potassium and chlorides. Amorphous silica was also included in the model as a potential secondary product of the transformation of calcium silicates present in steel slag.

### 5.2. Modelling Results—Interpretation of Geochemical Processes in the Slag-Water System

The interpretation of the inverse modelling results was based on the integration of three independent datasets: (i) quantitative XRD phase analysis, (ii) groundwater hydrochemistry and (iii) calculated mineral mass transfers obtained from PHREEQC. Consistency between these datasets was used as the principal criterion for evaluating the plausibility of the proposed reaction pathway.

PHREEQC inverse models are based on mass balance and assume the full availability of permitted mineral phases. In reality, however, only a fraction of the grains undergo a reaction, whilst the remainder remain virtually inert over a period of several years. The molar transfers calculated by PHREEQC should therefore be interpreted as apparent, balance-based net transfers, necessary to reproduce the observed change in the chemical composition of the solution, rather than as a direct measure of the actual rate of dissolution or precipitation of mineral phases in the slag material [20].

Taking kinetic constraints into account is therefore crucial both for the correct interpretation of geochemical modelling results and for assessing long-term changes in slag volume in road structures [27,28].

Inverse modelling yielded eight admissible minimal models that reproduce the observed evolution of the water’s chemical composition following contact with slag. All solutions were characterised by very similar residual error values and a maximum relative error of 5.9%. This indicates the internal consistency of the models obtained; however, it should be emphasised that these are solutions that are acceptable in terms of mass balance, rather than an unambiguous representation of the actual reaction pathway. Their interpretation therefore requires consideration of the limitations arising from the assumed set of phases, analytical uncertainty and the lack of information on the kinetics of the processes.

Although the inverse calculations yielded eight acceptable solutions satisfying the elemental mass balance, these should not be regarded as alternative geochemical scenarios but rather as mathematically equivalent solutions constrained by the specified analytical uncertainties. All accepted models consistently indicate the same principal reaction pathway, involving the dissolution of the primary Ca-rich slag phases accompanied by the precipitation of secondary carbonate, hydroxide and sulphate minerals. The differences between individual solutions concern mainly the magnitude of the calculated phase transfers, whereas the direction of the geochemical processes remains unchanged. This high degree of consistency indicates that the proposed geochemical evolution is robust despite the inherent non-uniqueness of inverse modelling. Furthermore, the inverse calculations were constrained by the experimentally determined mineral assemblage identified by XRD and SEM-EDS analyses, thereby reducing the range of geochemically plausible solutions and increasing confidence in the interpreted reaction pathway. Consequently, the discussion below focuses on the mineral reactions that are common to all accepted inverse models, as these represent the most reliable reconstruction of the groundwater–slag interaction.

All the models obtained indicated the dissolution of larnite (Ca_2_SiO_4_), merwinite (Ca_3_MgSi_2_O_8_), mayenite (Ca_12_Al_14_O_33_), halite (NaCl) and, in some variants, slag glass as well (Table 6).

Of the phases listed, larnite and merwinite play the most important role, as they are the main sources of calcium and magnesium in the solution. These minerals are among the most common constituents of steelmaking slags and are regarded as the key phases controlling the chemical composition of the leachate produced during their weathering [25].

Of particular significance is the dissolution of larnite, the mass transfer of which reached approximately 0.01 mol (Table 6). Larnite is one of the most reactive calcium silicates found in slags. On contact with water, it releases calcium ions and contributes to the high alkalinity of the solution. Numerous experimental studies have shown that it is precisely the dissolution of calcium silicates that constitutes one of the main mechanisms responsible for the rapid increase in the pH of slag leachates to values exceeding 11–12 [25,29]. It has also been shown that the dissolution reactions of Ca-rich silicates and aluminosilicates proceed more slowly in real systems than in thermodynamic models, partly due to the limited availability of reactive surface area [30,31].

Merwinite also plays a significant role, with its mass transfers reaching values comparable to those of larnite (Table 6). The results obtained indicate that the dissolution of this phase is the main source of the observed increase in magnesium concentration. Mineralogical studies of AOD and EAF slags demonstrate that merwinite is one of the commonly occurring magnesium-bearing silicate phases, and its dissolution is a significant source of Mg released into solutions during slag weathering [25,32].

Mayenite was present in almost all models, although its mass transfers were significantly lower than those of larnite and merwinite (Table 6). However, the significance of this phase stems not from the quantity of material dissolved, but from its very high reactivity. Mayenite undergoes rapid hydration, and even small amounts of this mineral can significantly influence the increase in alkalinity and the release of calcium and aluminium into the solution [33,34].

In some of the models, it was also necessary to take into account the dissolution of slag glass. The presence of this phase indicates that the observed chemical evolution was not controlled solely by crystalline phases (Table 6). The amorphous glassy matrix formed during the rapid cooling of slag is characterised by greater reactivity than most crystalline phases due to the absence of an ordered crystalline structure and higher surface energy [17]. For this reason, it may constitute a significant source of calcium, magnesium, aluminium and silicon in the initial stage of water’s interaction with the slag material.

The greatest positive mass transfer was observed for halite (Table 6). This process directly explains the significant increases in the concentrations of sodium and chlorides between the initial and final solutions. Due to halite’s very high solubility, its dissolution occurs almost immediately upon contact with water. The fact that this phase is present in all eight models is related not so much to the presence of readily soluble salts in the material under investigation, but rather to an external source. The water samples for the tests were collected during the winter; hence, the presence of halite may be linked to the use of salt in road maintenance, i.e., for gritting the streets. In this sense, halite should be treated in the model primarily as a balancing component representing an external influx of Na and Cl into the system, rather than as a primary mineral phase of the slag controlling its geochemical properties. The elevated sodium and chloride concentrations observed in some sampling points should be interpreted in the context of seasonal conditions. Although the inverse modelling indicates that the principal geochemical transformations of the slag are controlled primarily by dissolution of Ca-rich mineral phases and subsequent precipitation of secondary minerals, the contribution of externally supplied Na and Cl may vary throughout the year depending on meteorological conditions and road maintenance practices. Therefore, the hydrochemical conditions described here represent the investigated period rather than the full annual variability in the groundwater system. Future multi-season monitoring would enable a more comprehensive assessment of the temporal evolution of groundwater chemistry and the long-term weathering behaviour of steel slag under field conditions.

Inverse models indicate that the dissolution of the primary slag constituents was accompanied by the precipitation of secondary mineral phases, primarily amorphous silica, calcite, ettringite, kalicinite and brucite (Table 6). Minor amounts of poorly crystalline C–S–H may also have formed; however, this phase was not included in the inverse model because it was not unambiguously identified by XRD, and its variable Ca/Si ratio and water content preclude the assignment of a unique stoichiometry.

The largest negative mass transfer was observed for amorphous silica (Table 6). This result indicates that the amount of silicon released during the dissolution of larnite and merwinite was greater than the amount remaining in solution at equilibrium. The excess dissolved silica was therefore removed by the precipitation of a weakly ordered amorphous phase. This process is commonly observed during the weathering of steelmaking slags and constitutes one of the fundamental mechanisms regulating the concentration of silicon in leachates [17,25].

Calcite precipitation occurs in all models (Table 6). This process is a consequence of the high pH and high availability of calcium in the solution. Under such conditions, the system rapidly becomes supersaturated with respect to CaCO_3_, leading to carbonation [24]. Carbonation is one of the best-documented processes occurring during the ageing of steelmaking slags and plays a significant role in stabilising their environmental impact [32].

Ettringite precipitation was observed in all model solutions (Table 6). Ettringite is stable in an environment rich in calcium, aluminium and sulphates at high pH [33]. Its formation is one of the fundamental mechanisms of sulphate immobilisation in both cementitious materials and steelmaking slags [33,35]. The presence of ettringite provides a good explanation for the significant decrease in sulphate concentration observed between solutions 1 and 2. At the same time, this process leads to a reduction in the mobility of aluminium and some of the calcium.

The fact that ettringite is present in all eight models suggests that the secondary precipitation of sulphates was one of the key processes responsible for determining the final chemical composition of the solution under investigation.

In some of the models, brucite precipitation was also observed (Table 6). The formation of this phase is favoured very high pH and is often interpreted as an indication of an advanced stage of weathering in magnesium-rich materials [17]. Although the pH of the bulk S5 water sample was 12.49, locally higher pH and Mg^2+^ activity at the surfaces of dissolving slag grains may have created microenvironments favourable for brucite precipitation. The presence of this phase in some models therefore suggests that part of the magnesium released during merwinite dissolution reached supersaturation with respect to brucite. The simultaneous precipitation of calcite, ettringite, amorphous silica and, locally, brucite indicates that the investigated system was gradually moving towards greater geochemical stability through the removal of excess constituents from the solution.

Portlandite did not appear in any of the eight models obtained, despite its presence having been confirmed in the XRD analyses. This phase was included in the model as a possible secondary product; however, it did not appear in the resulting solutions as a required molar transfer. The absence of portlandite in the models does not negate its mineralogical presence, but indicates that, given the specified solution compositions, phase composition and the assumed uncertainty of 0.059, its contribution is not necessary to describe the final chemical balance of the solution. The discrepancy between the presence of portlandite in the XRD analysis and the absence of its unambiguous transfer in inverse modelling can be explained by the difference between the mineralogical composition of the solid material and the solution balance. Portlandite may have formed locally as a result of the hydration of calcium phases, particularly larnite, but its effect on the balance may have been masked by simultaneous carbonation and calcite precipitation. In such a system, portlandite occurs as a phase that is indeed present in the slag, but not necessarily as the phase controlling the final difference in solution composition.

The results of inverse modelling allow for the proposal of a two-stage model of the geochemical evolution of the system under study. The first stage involves the dissolution of reactive slag components—primarily larnite, merwinite, mayenite, halite and, locally, slag glass—which dominated the balance and led to increases in pH, alkalinity and the concentrations of Ca, Mg, Na and Cl in solution. The second stage involves the precipitation of secondary mineral products, mainly amorphous silica, calcite and ettringite, which are responsible for limiting the mobility of Si, SO_4_^2−^ and part of the Ca. It should be emphasised, however, that this division is of a paragenetic-balance nature, rather than directly kinetic.

The quantitative phase composition determined by XRD provides an important framework for interpreting the calculated phase transfers. Larnite (18.6 wt.%) and merwinite (13.7 wt.%) constitute the principal reactive calcium-bearing silicate phases in the investigated slag and therefore represent the dominant potential sources of Ca released to the aqueous phase. Their calculated dissolution is consistent with the pronounced increase in calcium concentration observed in groundwater after contact with the slag and with the subsequent precipitation of calcite, ettringite and brucite identified by inverse modelling. Although slag glass represents the largest individual component of the material (26.0 wt.%), its contribution should be interpreted primarily as a long-term source of Si and Al released during progressive weathering rather than as the principal source of calcium. Thus, the inverse modelling results are in good agreement with both the experimentally determined phase composition and the observed hydrochemical evolution of the groundwater–slag system.

Although the absolute amounts of mineral dissolution cannot be directly related to the bulk phase percentages determined by XRD, the consistency between the abundance of the principal reactive phases, the observed hydrochemical changes and the calculated molar transfers provides independent support for the proposed reaction pathway.

The high agreement among all eight inverse models indicates that the reaction scheme presented constitutes the most likely description of the geochemical evolution of the water–slag system under study. The results obtained are fully consistent with the current state of knowledge regarding the leaching, carbonation and secondary mineralisation processes occurring in steel slags [17,24,25].

It should be emphasised that the inverse model describes only the mass balance, and not the actual kinetics of mineral processes. This means that the dissolution or precipitation of a given phase in the model does not necessarily reflect the intensity of these processes in the actual slag material, where some phases may be less reactive or only partially available. The mechanism limiting the reaction rate is the formation of secondary mineral coatings, such as brucite, calcite, chalcedony or C-S-H gels. These layers reduce the diffusion of water into the interior of the grains, leading to a gradual slowing down of the reaction over time [36]. This effect is particularly pronounced under high pH conditions, which are characteristic of slag–water systems.

The consistency of the models obtained with the XRD data allows them to be regarded as balanced representations of possible successive reaction states of the slag–solution system. The proposed sequence involves: early hydration of larnite and the supply of Ca-Si, partial carbonation of Ca to calcite and the fixation of sulphates into ettringite, followed by an increasing proportion of merwinite as a source of Ca-Mg-S, the precipitation of brucite as a secondary reservoir of Mg, and the possible contribution of slag glass as an additional source of constituents. The presence of portlandite in the mineralogical record can, in this context, be interpreted as the result of the local or stage-specific retention of Ca(OH)_2_, even though this phase is not dictated by the final solution balance.

This interpretation is paragenetic–evolutionary rather than kinetic: the models do not establish the chronological order of the reactions but identify phase combinations consistent with the chemical balance and mineralogical observations. However, their interpretation is limited by the inherent non-uniqueness of inverse modelling, the unconstrained water-to-rock ratio and contact time, the lack of an independent dataset for validation, and the dependence of the calculated mass transfers on the selected phases and their stoichiometry; consequently, the possible formation of minor or poorly crystalline phases not identified by XRD cannot be excluded.

Although the slag aggregate used in the road construction was a commercial material intended to meet the applicable technical and environmental requirements, the absence of Cr, V, Mo and Zn determinations in groundwater limits the present assessment to major-ion hydrogeochemistry and precludes evaluation of the field-scale mobility of these trace elements.

## 6. Summary and Conclusions

The research carried out has shown that contact between groundwater and aggregate produced from steelmaking slag leads to marked changes in the chemical composition of the water infiltrating through the slag material. The most significant changes include increases in pH, alkalinity and the concentrations of Ca, Mg, Na and Cl, confirming active geochemical interaction within the water–slag system.

The results of mineralogical analyses and PHREEQC inverse modelling indicate that the main phases responsible for the alkalisation of the solution and the supply of constituents to the water were larnite, merwinite, mayenite and, locally, slag glass. Larnite was the primary source of calcium, whilst merwinite was a significant source of calcium, magnesium and silicon.

Inverse modelling made it possible to quantify the mass transfers of the mineral phases responsible for the observed change in water chemistry. The models obtained indicate that the dissolution of reactive primary phases was accompanied by the precipitation of secondary mineral products, mainly amorphous silica, calcite, ettringite and brucite.

The precipitation of calcite and ettringite indicates the significant role played by carbonation and the secondary binding of sulphates in stabilising the system. These processes limited the mobility of some constituents, particularly Ca, SO_4_^2−^ and Al, and influenced the final chemical composition of the water draining through the slag aggregate.

The largest positive mass transfer observed for halite suggests that the increases in Na and Cl concentrations were most likely linked to an external influx of these components, probably due to the use of road salt during the winter months. Halite should therefore be interpreted as a balancing component representing the influx of Na-Cl into the system, rather than as a diagnostic mineral phase of the slag.

The absence of portlandite as a required molar transfer in inverse models, despite its presence in mineralogical studies, indicates that this phase did not control the final chemical balance of the solution. Portlandite may have formed locally or in stages, but its effect on the chemical balance was probably masked by simultaneous carbonation and calcite precipitation.

The slag aggregate under investigation is not a geochemically inert material when in contact with groundwater; however, the simultaneous processes of carbonation and secondary mineralisation indicate the gradual stabilisation of the water–slag system.

From an engineering perspective, the results demonstrate that the long-term performance of steel slag aggregates cannot be assessed solely on the basis of their initial physical and mechanical properties. Their geochemical reactivity under site-specific hydrogeological conditions should also be considered, particularly where prolonged groundwater contact may occur. The observed dissolution of reactive calcium-bearing phases and the formation of secondary minerals indicate that groundwater chemistry may evolve over time, potentially influencing both the hydraulic behaviour of the ballast layer and the quality of drainage water. Although the investigated slag remained geochemically active, the simultaneous precipitation of secondary carbonate and hydroxide phases suggests a gradual tendency towards the geochemical stabilisation of the system. These findings highlight the importance of integrating hydrogeological conditions, groundwater chemistry and mineralogical characteristics into the environmental assessment and design of road structures incorporating steel slag aggregates, especially in areas with high groundwater tables or where prolonged water–slag interaction is expected.

## Figures and Tables

**Table 1:** Physicochemical parameters and chemical composition of waters collected from piezometers, wells, stormwater inlets and a drainage ditch receiving waters from the waterproofing system. Explanatory notes: P—piezometers; S—wells; W—stormwater inlets; R1—surface-water sample from the ditch discharging drainage water from the waterproofing system, together with precipitation water and pavement-dewatering water, released into the adjacent river.

Quality Determinant	Unit	Sampling Point						
		P2	P3	P6		P7	S1	S2
Reaction	pH	6.95	6.96	6.81		6.94	6.59	6.96
Dry residue	mg/dm^3^	570	729	559		488	987	658
Mineralization	mg/dm^3^	746.46	902.50	741.54		664.46	1242.56	889.22
Alkalinity	mval/dm^3^	5.8	5.9	6		5.8	8.4	7.6
Total hardness	°n	20.19	26.36	20.19		20.19	33.09	26.92
Temporary hardness	°n	16.26	16.54	16.82		16.26	23.55	21.31
Permanent hardness	°n	3.93	9.82	3.36		3.93	9.53	5.61
Calcium hardness	°n	14.02	16.26	13.46		12.90	19.07	14.58
Magnesium hardness	°n	6.17	10.10	6.73		7.29	14.02	12.34
HCO_3_^−^	mg HCO_3_/dm^3^	352.91	359.00	365.08		352.91	511.11	462.44
Cl^−^	mg Cl/dm^3^	85.20	113.60	85.20		49.70	113.60	71.00
SO_4_^2−^	mg SO_4_/dm^3^	149.00	226.00	94.00		84.00	243.00	192.00
Ca^2+^	mg Ca/dm^3^	100.20	116.23	96.19		92.18	136.27	104.21
Mg^2+^	mg Mg/dm^3^	26.75	43.78	29.18		31.62	60.80	53.50
K^+^	mg K/dm^3^	12.00	20.00	6.90		6.70	14.00	10.60
Na^+^	mg Na/dm^3^	86.86	89.13	67.86		35.67	102.90	84.78
Fe^3+^/Fe^2+^	mg Fe/dm^3^	0.08	0.075	0.078		0.294	0.209	0.340
Mn^2+^	mg Mn/dm^3^	0.10	0.10	0.38		0.07	0.05	0.33
Al^3+^	mg Al/dm^3^		0.0001					
Si^4+^	mg Si/dm^3^		0.0001					
**Quality** **Determinant**	**Unit**	**Sampling Point**						
		**S3**	**S4**	**S5**	**S7**	**W2**	**W3**	**R1**
Reaction	pH	6.72	12.76	12.49	12.96	8.18	7.17	12.45
Dry residue	mg/dm^3^	758	1177.00	2371.00	1768.00	612	658	866
Mineralization	mg/dm^3^	964.88	1770.26	3919.55	2851.08	700.23	837.50	1249.34
Alkalinity	mval/dm^3^	6.8	19.5	50.9	35.6	2.9	5.9	12.6
Total hardness	°n	31.40	83.00	155.90	120.01	18.51	29.72	56.08
Temporary hardness	°n	19.07	54.68	142.72	99.82	8.13	16.54	35.33
Permanent hardness	°n	12.34	28.32	13.18	20.19	10.37	13.18	20.75
Calcium hardness	°n	25.24	50.47	128.98	80.76	8.41	16.82	32.53
Magnesium hardness	°n	6.17	32.53	26.92	39.26	10.09	12.90	23.55
HCO_3_^−^	mg HCO_3_/dm^3^	413.76	1186.51	3097.10	2166.15	176.46	359.00	766.67
Cl^−^	mg Cl/dm^3^	134.90	631.90	1739.50	1157.30	191.70	120.70	369.20
SO_4_^2−^	mg SO_4_/dm^3^	248.00	140.00	20.00	31.00	199.00	168.00	121.00
Ca^2+^	mg Ca/dm^3^	180.36	360.72	921.84	577.15	60.12	120.24	232.46
Mg^2+^	mg Mg/dm^3^	26.75	141.06	116.74	170.24	43.78	55.94	102.14
K^+^	mg K/dm^3^	18.00	14.00	18.00	23.00	15.00	14.00	14.00
Na^+^	mg Na/dm^3^	92.69	234.73	1014.85	583.13	125.28	41.91	117.90
Fe^3+^/Fe^2+^	mg Fe/dm^3^	1.411	0.050	0.070	0.364	0.168	0.050	0.111
Mn^2+^	mg Mn/dm^3^	0.24	0.05	0.05	0.05	0.05	0.08	0.05
Al^3+^	mg Al/dm^3^			0.0002				
Si^4+^	mg Si/dm^3^			0.0002				

**Table 2:** Quantitative phase composition of the investigated steel slag (in %).

Phase		Chemical Formula	Share (%)
Primary phases	Wüstite	FeO	9.5
	Srebrodolskite	Ca_2_((Fe_1.592_Al_0.408_)O_5_)	14.9
	Brownmillerite	Ca_2_((Fe_1.45_Al_0.55_)O_5_)	3.8
	Mayenite	(CaO)_12_(Al_2_O_3_)_7_	3.0
	Larnite	Ca_2_(SiO_4_)	18.6
	Merwinite	Ca_3_Mg(SiO_4_)_2_	13.7
	Slag glass	Ca_0.50_Mg_0.20_AlSiFe_0.05_O_4.25_	26.0
Secondary phases	Si-rich alteration material *	SiO_2_-rich	4.5
	Lime	CaO	1.7
	Calcite	CaCO_3_	0.8
	Portlandite	Ca(OH)_2_	1.8
	Brucite	Mg(OH)_2_	1.0
	Ettringite	Ca_6_Al_2_(SO_4_)_3_(OH)_12_•26(H_2_O)	0.7
Total			100.0

*—The Si-rich alteration material is interpreted as a secondary amorphous or poorly crystalline product related to the alteration of calcium silicates; it is not treated here as a separate crystalline phase.

**Table 3:** Chemical compositions, calculated atomic proportions, and average empirical formula of selected slag-glass micro-areas analysed by EDS (in wt.%).

Chemical Compound	EDS Analytical Point (from Figure 5)						Average
	20	21	25	26	27	28	
CaO	18.54	17.90	19.80	19.00	19.02	18.14	18.73
MgO	5.04	6.25	4.92	4.52	5.13	5.46	5.22
Al_2_O_3_	35.01	31.15	35.01	35.11	33.22	33.42	33.82
SiO_2_	39.30	41.23	38.61	39.48	40.45	39.56	39.77
FeO	2.11	2.68	1.66	1.89	2.18	3.02	2.26
Total	100.00	99.21	100.00	100.00	100.00	99.60	99.80
Element	number of atoms						
Ca	0.50	0.48	0.53	0.51	0.51	0.49	0.50
Mg	0.19	0.24	0.18	0.17	0.19	0.21	0.20
Al	1.03	0.93	1.04	1.04	0.98	0.99	1.00
Si	0.98	1.04	0.97	0.99	1.01	1.00	1.00
Fe	0.04	0.06	0.03	0.04	0.05	0.06	0.05
O	4.25	4.25	4.25	4.25	4.25	4.25	4.25
Average empirical formula	Ca_0.50_Mg_0.20_AlSiFe_0.05_O_4.25_						

**Table 4:** Chemical composition of the input solutions used in the model.

Parameter	Unit	Solution 1	Solution 2	Interpretation
pH	–	6.96	12.49	alkalization due to dissolution of calcium-bearing phases
Alkalinity	mmol/L	5.883	50.755	increase in system alkalinity
Ca	mmol/L	2.90	23.0	dissolution of larnite and merwinite
Mg	mmol/L	1.80	4.802	dissolution of merwinite
Na	mmol/L	3.877	44.143	dissolution of halite
Cl	mmol/L	3.205	49.069	dissolution of halite
SO_4_^2−^ (S(6))	mmol/L	2.353	0.208	precipitation of ettringite
Al	mmol/L	0.0001	0.0002	dissolution of aluminium-bearing phases
Si	mmol/L	0.0001	0.0002	dissolution of silicates + secondary precipitation of SiO_2_ (am)

**Table 5:** Mineral phases included in the inverse modelling and their role in the system.

Phase	Status	Character	Role in the Geochemical Process
Larnite	dis	Primary slag phase	Primary Ca_2_SiO_4_—highly reactive calcium silicate
Merwinite	dis		Primary calcium-magnesium silicate of slag—source of Ca, Mg and Si
Mayenite	dis		Ca-Al slag phase—releases Al and Ca
Brownmillerite	dis		Ca-Fe-Al phase—source of Ca and Fe
Srebrodolskite	dis		Primary Ca_2_Fe_2_O_5_ phase—source of Ca and Fe
Wüstite	dis		Primary Fe oxide—important source of Fe in solution
Slag glass	dis		Primary glassy phase
Halite	dis	Neutral/ external	NaCl—external input of Na and Cl
Kalicinite	pre		KHCO_3_—external input of K and carbonate
Calcite	pre	Secondary phase (reaction product)	Secondary CaCO_3_—main Ca-C buffer
Ettringite (Fe)	pre		Secondary Ca-Fe sulfate—reaction product with sulfates
Brucite	pre		Secondary Mg(OH)_2_ phase—may precipitate at high pH
Portlandite	pre		Secondary Ca(OH)_2_—product of calcium silicate decomposition
Lime	pre		Hydration of CaO—secondary mineral associated with a strongly alkaline environment
Amorphous_silica	pre		Amorphous silica—secondary product controlling SiO_2_ activity

**Table 6:** Range of molar transfers for mineral phases obtained in eight inverse models.

Phase	Mass Transfer (mol)	Geochemical Significance
Larnite	+0.0097 to +0.0104	main source of Ca, alkalisation and pH increase
Merwinite	+0.0027 to +0.0097	source of Ca and Mg
Mayenite	+1.02 × 10^−4^	source of Ca and Al
Brownmillerite	+5.798 × 10^−7^	subordinate source of Fe and Al
Srebrodolskite	+4.34 × 10^−7^	trace contribution of Fe
Wüstite	+7.24 × 10^−5^ to +8.64 × 10^−7^	source of Fe
Slag glass	+0.0014	source of Ca, Mg, Si and Al
Halite	+0.0433	source of Na and Cl
Kalicinite	−4.92 × 10^−5^	secondary binding of K
Calcite	−0.0065	carbonation
Ettringite(Fe)	−7.15 × 10^−4^	immobilisation of SO_4_^2−^
Brucite	−0.0070	secondary binding of Mg
Portlandite	0	not required by the models
Amorphous_silica	−0.0158 to −0.0208	removal of excess Si

## Data Availability

The original contributions presented in this study are included in the article. Further inquiries can be directed to the corresponding author.
